# ﻿An account of the genus *Cistanche* (Orobanchaceae) in Iraq and taxonomic considerations in the Middle East

**DOI:** 10.3897/phytokeys.238.116470

**Published:** 2024-02-28

**Authors:** Majed Aldughayman, Chris J. Thorogood, Abdulridha A. A. Al-Mayah, Julie A. Hawkins

**Affiliations:** 1 School of Biological Sciences, Health and Life Sciences Building, University of Reading, Whiteknights, Reading, RG6 6EX, UK University of Reading Reading United Kingdom; 2 Department of Biology, University of Oxford, South Parks Road, Oxford, OX1 3RB, UK University of Oxford Oxford United Kingdom; 3 Oxford University Botanic Garden, Oxford, OX1 4AZ, UK Oxford University Botanic Garden Oxford United Kingdom; 4 Department of Ecology, College of Science, University of Basrah, Basrah, Iraq University of Basrah Basrah Iraq

**Keywords:** Nomenclature, parasitic plant, speciation, taxonomy

## Abstract

Species limits in the genus *Cistanche* are poorly understood, despite the plants’ long history of use in traditional herbal medicine and food across their range. Here we present a taxonomic account for the genus *Cistanche* in Iraq, where several taxa have been reported, most of them doubtfully. Using herbarium specimens, images of living material, and taxonomic literature, we found evidence of only one species occurring with certainty in Iraq: *Cistanchetubulosa*. We found no evidence for the occurrence of other *Cistanche* species in Iraq, including a putative new entity reported for the region. Our work highlights inconsistencies in the literature, and underscores the importance of examining multiple stable characters for delimiting species in the genus *Cistanche*.

## ﻿Introduction

Iraq has a rich flora with an estimated 3300 species owing to a convergence of phytogeographic regions and varied climate and topography (Zohary 1973; Ghazanfar and McDaniel 2016). Until the mid-twentieth century, there were no checklists or Floras for this young state (Frodin 2001; Ghazanfar and McDaniel 2016). In the 1950s, the first national checklist, The Flora of Iraq and its Phytogeographical Subdivision, was written by Michael Zohary (Frodin 2001; Ghazanfar and McDaniel 2016). In 1964 Karl-Heinz Rechinger published the Flora of Lowland Iraq (Rechinger 1964a) with contributions by multiple authors, including a treatment for the Orobanchaceae by H. Schiman-Czeika. A year later, the Flora of Iraq began as a collaborative project between the Royal Botanic Gardens, Kew and the Ministry of Agriculture, Baghdad (Frodin 2001; Ghazanfar and McDaniel 2016). From this project, volumes 1, 2 and 3, and volume 4 parts 1 & 2, 8 and 9 were published; volumes 5, 6 and 7 remained unpublished due to political instability in the region, and the project was suspended in the 1980s (Guest and Townsend 1966; Townsend and Guest 1966–1985; Frodin 2001; Ghazanfar and McDaniel 2016). Then in the 2010s, the Flora of Iraq project resumed as a collaboration between the Royal Botanic Gardens, Kew and the Ministry of Agriculture, Baghdad, in 2013, Vol. 5(2) was published and vol. 5(1) is in press. Volumes 6 and 7 which cover around 900 species are in progress (Ghazanfar and McDaniel 2016). The family Orobanchaceae has been written for this ambitious project but remains, as yet, unpublished.

The genus *Cistanche* Hoffmanns. & Link (family Orobanchaceae), was first identified officially in 1799 by Ventenat under the name Orobanchoideae. A decade later, the genus *Cistanche* was described by Hoffmannsegg and Link (1813). The latest monograph for the family Orobanchaceae was published in 1930 by Beck-Mannagetta (Beck-Mannagetta 1930). He divided *Cistanche* species into four sections based on calyx and bracteole morphology; however the first comprehensive phylogeny for the genus *Cistanche* revealed that none of these sections are monophyletic, with the exception of C.sect.Subcistanche (Ataei et al. 2020). Moreover, this phylogeny revealed that *Cistanche* species form four well-supported, geographically differentiated clades which they described as the Northwest African Clade, Southwest Asian Clade, Widespread Clade and East Asian Clade. The East Asian Clade is the only clade that corresponds to a previously recognized taxonomic section (C.sect.Subcistanche). Despite progress in understanding the evolutionary relationships in the genus, a well-sampled phylogeny substantiated with detailed morphological and ecological data are absent, and species limits remain confused and uncertain.

*Cistanche* is a holoparasite that lacks vegetative traits traditionally used in taxonomy, including functional leaves and roots. The poor condition of herbarium specimens — particularly type specimens — has generated confusion in identification. Here we examine herbarium specimens, images of living material, and taxonomic literature to produce the first robust review of the genus *Cistanche* in Iraq, which will inform the treatment for the Flora, and other treatments for the genus in the Middle East.

## ﻿Material and methods

### ﻿Study species

We reviewed the names used for *Cistanche* in Iraq and neighbouring countries. The sources that were used to identify species of *Cistanche* putatively in Iraq, and the species in their accounts, were as follows:

### ﻿Treatments including Iraq

1. Flora of Lowland Iraq (Rechinger 1964a). Rechinger’s account referred to two species, *C.tubulosa* (Schenk) Wight ex Hook.f. and *C.salsa* (C.A.Mey.) Beck. He presented a key discriminating the species by height, pubescence and anther cell shape. *C.tubulosa* was described as a larger plant, 60–100cm, glabrous, and with obtuse anther cells. *C.salsa* was reported to grow up to 40 cm, lanate to glabrescent, and with acuminate anther cells. Five specimens of *C.tubulosa* and one of *C.salsa* were examined.
2. Flora Iranica (Rechinger 1964b), in which Iran, Persia, Afghanistan, parts of West-Pakistan, Iraqi Kurdistan, Azerbaijan and Turkmenistan listed *C.ridgewayana* Aitch. & Hemsl., *C.fissa* C.A.Mey.) Beck, *C.salsa*, *C.eremodoxa* Bornm., *C.laxiflora* Aitch. & Hemsl., *C.tubulosa* and *C.flava* (C.A.Mey.) Korsh. There were no reports of *Cistanche* species in autonomous Iraqi Kurdistan.
3. The Flowering Parasitic Plants of Iraq (Karim 1978) cites only *C.tubulosa*. Karim (1978) was aware of seven plants and cited three host species.
4. In an unpublished PhD thesis that included a monographic treatment of genus *Cistanche*, Ataei (2017) cited two species in the exsiccatae for Iraqi specimens: *C.tubulosa* and *C.flava*. She referred to the *C.salsa* specimen cited by Rechinger (1964a), considering it to be a misidentified specimen of *C.ambigua* (Bunge) Beck. In the treatment she also referred to an, as yet, unpublished species found in Iraq, but no specimen from Iraq was cited.
5. In an unpublished thesis entitled taxonomical and ecological study of parasitic plants of Iraq, Al-Asady (2017) cited only one species, *C.tubulosa* to occur in Iraq. The *C.salsa* specimen of in the Flora of Lowland Iraq (Rechinger 1964a) was considered to be a misidentified specimen of *C.tubulosa*.
6. The Ecology and flora of Basrah (Al-Mayah et al. 2016) cites only *C.tubulosa*. Al-Mayah et al. (2016) refer to ‘Zib AL-Zumal’, ‘Thenun AL-Jinn’ and ‘Halook’ as common names for *C.tubulosa* in Iraq. They also cite *Haloxylonsalicornicum* (Moq.) Bunge ex Boiss. (Amaranthaceae) and *Zygophyllumpropinquum* Decne. (Zygophyllaceae) as host species.
7. In their book on parasitic plants, Al-Mayah and Al-Asady (2022) stated only *C.tubulosa* to occur in Iraq. They cite the following hosts: *Haloxylonsalicornicum* (listed under its synonym: *Hammadasalicornica* (Moq.) Iljin) (Amaranthaceae), *Zygophyllumpropinquum* (listed under its synonym: *Tetraenapropinqua*) (Zygophyllaceae) and *Capparisspinosa* L. (Capparaceae).

### ﻿Treatments of neighbouring countries

1. The Flora of Syria, Palestine and Sinai (Post 1932) covers the region from the eastern Mediterranean seaboard to the Syrian Desert. This flora recorded *C.lutea* (Desf.) Hoffmanns. & Link, *C.tubulosa* and *C.salsa*. In the descriptions, *C.lutea* and *C.tubulosa* are described as glabrous and *C.salsa* as lanate. Of the three species, only *C.lutea* is reported to occur in the desertic regions contiguous with Iraq.
2. In the Flora of Turkey (Davis 1982), *C.salsa* is the only species recorded. It is described as having densely lanate bracts and bracteoles, and a glabrous calyx and corolla. It was cited in three regions, two were in Inner Anatolia and one was in Kars city which is only 370 km from the Iraqi Kurdistan border. Therefore, *C.salsa* is a possible candidate species for the Flora of Iraq.
3. The Flora of Saudi Arabia (Migahid 1989) recorded two species, *C.phelypaea* (L.) Cout. (generally considered an Atlantic species) and *C.tubulosa*. Other species that are not included in the Flora have not been recorded for Saudi Arabia. These are *C.violacea* and *C.rosea* Baker (Foley 2004). The distribution of these species is narrow, *C.rosea* is distributed in the far south west and *C.violacea* in the northwest, far from the border with Iraq.
4. The Flora of Kuwait (Daoud 1985) records only *C.tubulosa*, cites *Sodarosmarinus* (Bunge ex Boiss.) Akhani (listed under its synonym: *Seidlitziarosmarinus* Bunge ex Boiss. (Amaranthaceae) as a host, and considers *C.tubulosa* var. *tomentosa* Hook.f.i.c as a Synonym.

### ﻿Examination of protologues, types and representative specimens

Protologues for *Cistancheeremodoxa*, *C.salsa*, *C.ridgewayana*, *C.fissa*, *C.laxiflora*, *C.flava*, *C.ambigua*, *C.lutea* and *C.phelypaea* — the species identified as putatively in Iraq and adjacent regions of neighbouring countries — were examined as well the description of the unpublished putative species *C.chabaharensis* (Ataei 2017). Type specimens of these species were examined when they were available; type specimens of *C.flava* and *C.tubulosa* were unavailable, and are believed to be missing. The Natural History Museum, London (NHM) and the University of Vienna (WU) collections were examined, but they held no Iraqi specimens. An extensive survey of three herbaria: Kew (K), Edinburgh (E) and the Natural History Museum of Vienna (W) and examination of herbarium images from the National Herbarium in Baghdad (BAG) retrieved 62 specimens from Iraq. Specimens from adjacent countries were also examined for comparison. Images of living material in Iraq and adjacent countries sourced from social media were all examined, and cross-referenced with herbarium specimens.

### ﻿Distribution mapping

**Figure 1.**
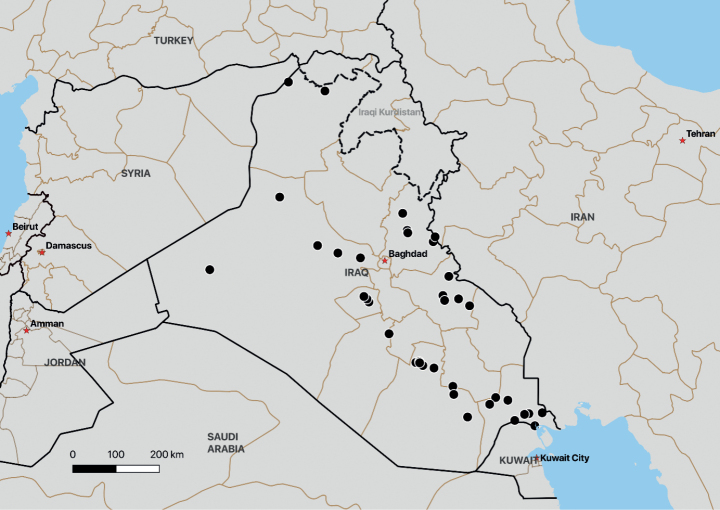
The locations of the material examined in Iraq.

Google Earth was used to create coordinate assumptions for those specimens for which true coordinates were unavailable (Fig. 1). Coordinates were exported to QGIS 3.4 with layers from the Natural Earth Quick Start Kit. Host species were recorded from specimen labels or based on identifications of host plant material mounted on the same sheet as the specimen. In the absence of excavation of host-parasite connections, the identity of the host was considered tentative (the parasite can appear some distance above ground from the host plant).

## ﻿Results and discussion

### ﻿*Cistanche* species in Iraq

A literature review of Iraq and of adjacent countries, and of other relevant taxonomic studies, identified 10 published names and one unpublished name for species putatively in Iraq and adjacent countries. Species in adjacent countries, especially in Iran including, *C.eremodoxa*, *C.ridgewayana*, *C.fissa*, *C.laxiflora* and *C.ambigua*, were excluded from this investigation as their distributions and morphology suggest they are unlikely to exist in Iraq. Of the other species, two Atlantic/western European taxa were excluded on the basis that they are exceptionally unlikely to occur in the Middle East: *C.lutea* and *C.phelypaea*. Although *C.lutea* was cited in the Flora of Syria, Palestine and Sinai, the description does not provide adequate detail of morphological characters to discriminate between this and other species, including *C.tubulosa*. *Cistanchephelypaea*, as circumscribed currently, is a primarily Atlantic, coastal species, frequent from southwest Portugal south to Macaronesia and the coast of Morocco. We consider the inclusion of *C.phelypaea* in the Flora of Saudi Arabia to be equivocal, and cited with insufficient detail to merit further examination. Moreno Moral et al. (2017) consider *C.lutea* and *C.phelypaea* to be morphologically distinct. Wood (1997) records the presence of *C.phelypaea* and *C.rosea* Bakir in Yemen and considered *C.tubulosa* a synonym of *C.phelypaea*. Author AM during his time in Yemen at the University of Taiz, in 2000–2002, observed a putatively distinct form of *Cistanche* across regions. It parasitized *Halothamnusbottae* Jaub. & Spach (syn. *Salsolabottae* (Jaub. & Spach) Boiss) (Amaranthaceae) (absent from Iraq) and was pure yellow, without purple pigmentation. Further investigation is required. *Cistancherosea*, which is widespread across the Arabian Peninsula, is readily distinguished by its deep rose-red corolla. Ataei et al. (2020) asserts that *C.lutea* and *C.phelypaea* do not occur in the Middle East. Both entities were excluded from further work for the treatment of the genus *Cistanche* in Iraq.

**Figure 2.**
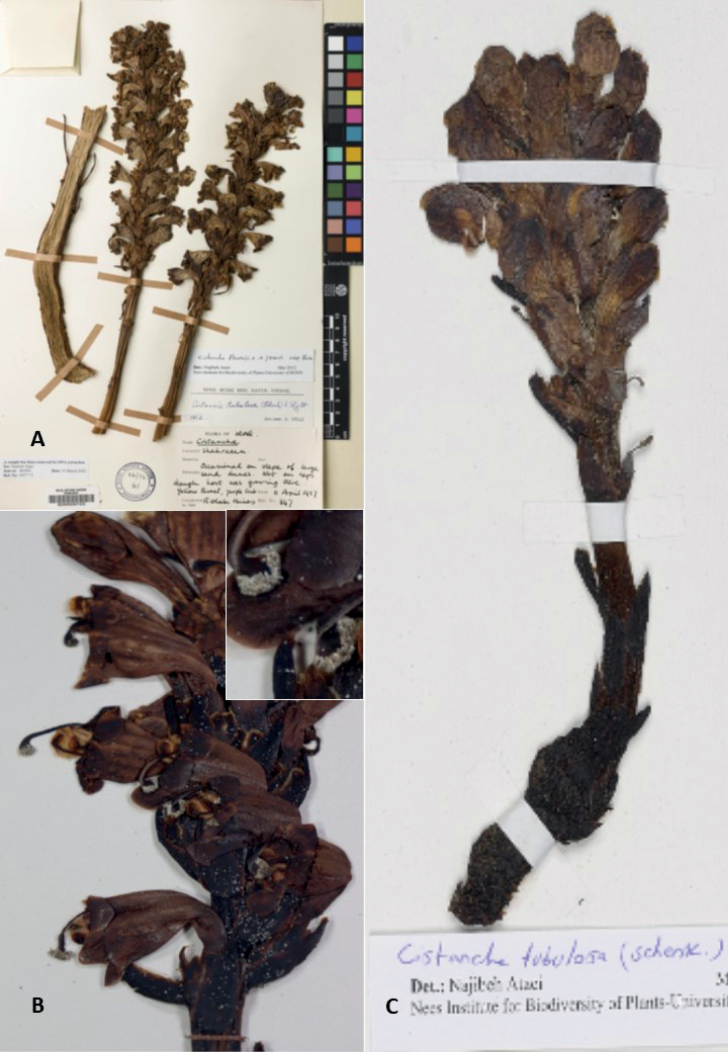
**A** herbarium specimen of *C.tubulosa* collected in Iraq, mistakenly identified as *C.flava***B** the holotype of putative species *C.chabaharensis*; note the woolly anther (inset) typical of *C.tubulosa***C** herbarium specimen of *C.tubulosa* in (W) collected in lowland of Iraq, mistakenly identified as *C.salsa*.

**Figure 3.**
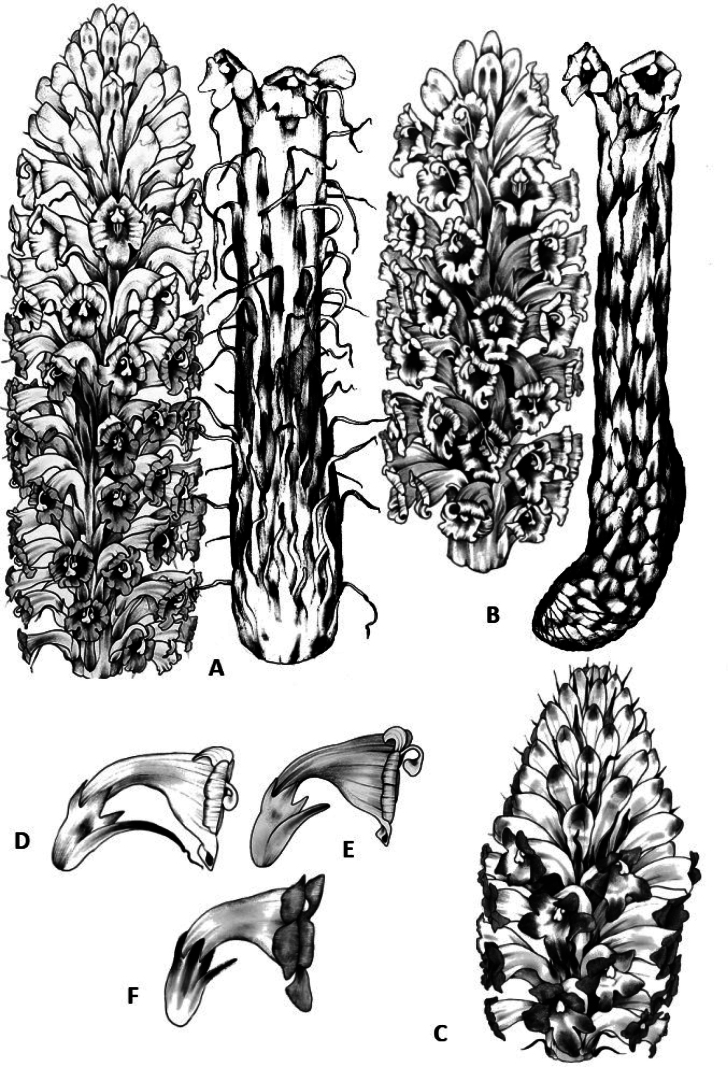
Illustrations of *Cistanche* species putatively found in Iraq and adjacent territories (inflorescences) **A***C.flava***B***C.tubulosa***C***C.salsa***D–F** corollas in profile of *C.flava*, *C.tubulosa* and *C.salsa*, respectively; note calyx and bract characteristics.

Finally, we considered *C.chabaharensis*, an as yet unpublished name referred to in the thesis of Ataei (2017) (Fig. 2B). This description placed focus on the glabrous, acute anthers; the key also described bract and bracteoles to possess non-sinuate margins, yellow corolla, and anther filaments glabrous at the base. However, on close examination of specimens cited by Ataei (2017) from Oman (McLeish, E00121976 E) and Iraq (Barkley & Abbas-Al-Ani, 6499 K), it was apparent both had woolly anthers that could not be distinguished from anthers of *C.tubulosa*, and that the bracts and bracteoles were sinuate. We consider it doubtful that this unpublished name represents a new entity found in Iraq; rather we believe this to be an entity that falls within the bounds of variability in *C.tubulosa* s.l. In summary, only three species warranted detailed investigation for the region in question: *C.tubulosa*, *C.salsa* and *C.flava* (Table 1; Fig. 3).

**Table 1.** Key morphological characters of *Cistanche* species putatively found in Iraq and adjacent territories.

Characters	*C.tubulosa*	*C.salsa*	*C.flava*
**Scales**	ovate- lanceolate, obtuse, glabrous, sinuate	ovate- lanceolate, obtuse, pilose on the outer side, scarious	long-lanceolate, obtuse, glabrous, scarious
**Bract**	ovate-lanceolate, glabrous, sinuate, equal or slightly longer than calyx	oblong-lanceolate, pilose on the outer side, longer than the calyx, entire	oblong-linear, glabrous, scarious, sinuate, twice long as the calyx or even as long as corolla
**Bracteole**	oblong- lanceolate, glabrous, sinuate, equal or shorter than calyx	linear-oblong, pilose on the outer side, entire, equal or slightly longer than calyx	oblong-linear, glabrous, sinuate, slightly shorter than calyx
**Calyx**	tubular, up to 1⁄2 total corolla length, 5 lobes, oblong to oblong-ovate, glabrous, sinuate	tubular, ca. 1/3 corolla length, 5 lobes, oblong, pilose on the outer side and at the margins, entire	tubular-campanulate, 5 lobes, oblong to oblong-ovate, glabrous, sinuate
**Corolla**	tubular-campanulate, 5 lobes, orbicular, glabrous, yellowish throughout or with violet limb (especially in bud)	campanulate, 5 lobes, orbicular, sparsely ciliate at the lobes, mauve and white	tubular-campanulate, 5 lobes, orbicular, glabrous, yellow to blue-violet
**Anther**	ovate, obtuse at ends, densely pilose	ovate, obtuse at base and slightly aristate at apex, densely pilose	ovate, obtuse at ends, densely pilose
**Stigma**	bilobate	bilobate	bilobate

### ﻿Morphology and evolutionary relationships

**Figure 4.**
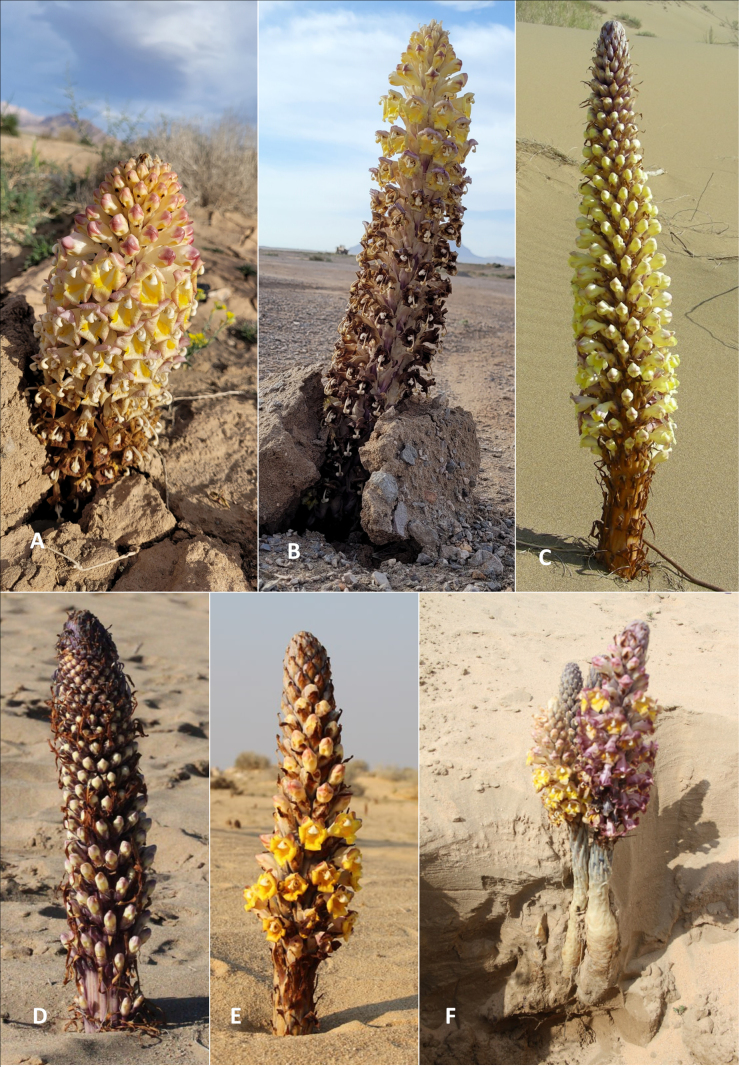
*Cistanche* species putatively found in Iraq and adjacent territories **A, B***C.tubulosa* (photographed in Iran) **C, D***C.flava* (photographed in Kazakhstan) **E, F***C.tubulosa* (photographed in Iraq; note excavated stem bases in **E**). Used with permission: photographs **A, B** by Sajad Alipour; photograph **C** by Lina Valdschmit; photograph **D** by Bobur Karimov.

Here we consider the three taxa identified to potentially co-occur in the region. *Cistanchesalsa* can be readily differentiated from *C.flava* and *C.tubulosa* by its hairiness; the latter two species are glabrous (see key). *Cistancheflava* is differentiated from *C.tubulosa* by its scarious, conspicuously long bracts which are twice as long as the calyx or even longer (Table 1; Figs 3A, D, 4C, D). Sánchez Pedraja et al. (2016) consider *C.flava* to be a synonym of *C.tubulosa*, however the distinct bract morphology is apparent both in living and dried material. Detailed, well-sampled investigations of both taxa are absent, and their distinction remains open to question; however recent molecular work provisionally supports their separation (Ataei et al. 2020).

The name *Cistanchetubulosa* s.l. is used from Africa and the Middle East to South and Central Asia and China, however as currently applied, the name refers to a widely distributed, polyphyletic group of plants (Aldughayman et al. 2024). In the most complete phylogeny (Ataei et al. 2020), specimens identified as *C.tubulosa* (or aff. *C.tubulosa*) were placed in a ‘widespread clade’, but four specimens identified as aff. *C.tubulosa* were nested in a separate clade sister to *C.flava*, along with other plants identified as *C.senegalensis* (an entity considered by Beck-Mannagetta to be closely related to *C.tubulosa*, but differentiated on the basis of subequal lower corolla lobes, oblong bracts and the absence of purple pigmentation). Additionally, two specimens identified as *C.tubulosa* are in a subclade which otherwise includes the Central Asian species, *C.laxiflora*. The type specimen of *C.tubulosa* is lost, and the correct application of the name *C.tubulosa* is yet to be clarified although it does seem to be misapplied to specimens in the *C.laxiflora* clade (Ataei et al. 2020; Aldughayman et al. 2024). Recently, a specimen collected from South Sinai near the type locality was designated a neotype (Aldughayman et al. 2024). This neotypification, alongside further phylogenetic work, is necessary to re-evaluate whether the name *Cistanchetubulosa* is a synonym for the name *C.tinctoria*, as has been proposed by Moreno Moral et al. (2017), and also to confirm whether the name *C.tubulosa* is the correct name for any Iraqi entity. To avoid compounding confusion, here we use the name *C.tubulosa*, consistent with most authors, until this re-evaluation is completed.

All material we examined from four Governorates: Karbala, Basrah, Muthanna and Wasit correspond to *C.tubulosa* (in its current, most widely-accepted circumscription). None of the material we examined pertained to either *C.salsa* or *C.flava*, based on our careful consideration of the traits emphasized above. Similarly, none of the specimens tentatively labelled *C.salsa* corresponded with that species either (Fig. 2C); indeed, we only found evidence of glabrous plants, ruling out the occurrence of *C.salsa* in Iraq based on the material available. Furthermore, material from Iraq identified as *C.flava* in a doctoral thesis (Ataei 2017) appears to have been identified in error (Fig. 2A): the unbroken bracts do not greatly exceed the calyx; we believe detached bracts may have caused confusion; phylogenetic analysis later confirmed the specimen in question nested with *C.tubulosa* (Ataei et al. 2020).

Our extensive investigation based on herbarium specimens revealed that *C.tubulosa* occurs in every Governorate of Iraq except for the Kurdistan Autonomous Region. This could be due to the wide distribution of potential hosts across three of four main ecological regions, namely the deserts west of the Euphrates River, Upper Mesopotamia and Lower Mesopotamia (Ghazanfar and McDaniel 2016; Hegazy and Doust 2016). *Cistanche* has not been recorded in the fourth ecological region, the northern highlands of Iraqi Kurdistan. This region is an extension of the great Eurasian alpine system, and not a typical habitat for *Cistanche* which is primarily desert-dwelling; moreover, hosts typically associated with *Cistanche* – shrubby Amaranthaceae such as *Haloxylon*, are absent from this ecological region (Ghazanfar and McDaniel 2016; Hegazy and Doust 2016). We conclude from this examination that despite multiple reports of various taxa, only one species occurs in Iraq with certainty: *Cistanchetubulosa*.

It is of note that the corolla colour of *C.tubulosa* varies with age and population, from pale lemon yellow, to deep orange-yellow, with varying levels of pink to violet pigmentation. Similarly, the height and stature vary from 15 cm to 130 cm depending on rainfall and, potentially, host species. The key below is based on our observations of multiple populations across the region.

### ﻿Key to *C.tubulosa* and potentially co-occurring taxa in Iraq and immediately adjacent regions

**Table:** 

1	Plants lanate to glabrescent; whitish, with purple pigmentation	***C.salsa***
–	Plants glabrous; cream to yellow with or without purple pigmentation	**2**
2	Bracts short: ovate-lanceolate, sinuate, equal to or scarcely exceeding the calyx	***C.tubulosa***
–	Bracts long and slender: oblong-linear, glabrous, scarious, sinuate, 2 x the calyx	***C.flava***

## ﻿Taxonomic treatment

### ﻿ Cistanche tubulosa

Taxon classificationPlantaeLamialesOrobanchaceae

(Schenk) R. Wight ex Hook.f., Fl. Brit. India, 4. 2:324. 1884.

468F9D06-D396-5112-8751-E50B4FB865EA

Phelypaea tubulosa Schenk (1840).

#### Notes.

A robust, thick, glabrous plant, (15)20–50(130) cm tall. Lower scales sinuate, imbricate, broadly lanceolate, up to 3 cm long. Upper scales sinuate, ovate-lanceolate, grey, 4–10 m long. Bracts deeply sinuate, ovate-lanceolate, equal or slightly exceeding the calyx, grey, 14–22 mm long. Bracteoles sinuate, oblong-lanceolate, equalling or shorter than calyx, grey, 2–3 mm long. Calyx tubular, pentamerous, usually 1/2 the corolla length, with lobes subequal or one slightly shorter, oblong, obtuse. Corolla tubular-campanulate, pentamerous, lemon yellow to deep yellow, often with violet limb, 34–52 mm long, lobes equal, rounded. Stamens didynamous, epipetalous, densely woolly at the base. Anthers cordate, rounded at the base and acute at the apex, densely woolly. Ovary ovate. Style cylindrical, oblique. Stigma bilobate. Fruit a splitting capsule. Seeds small, black and numerous.

#### Habitats.

Dunes, gravel substrates, mudstone, or seasonally arid saline habitats.

#### Hosts.

*Haloxylonsalicornicum* (Moq.) Bung, *Capparisspinosa*, *Zygophyllumpropinquum* Decne (syn. *Tetraenapropinqua*), *Tamarix* spp., *Salsola* spp.

#### Possible hosts.

*Ephedra* spp., *Limonium* spp., *Anabasis* spp.

#### Specimens examined.

**Iraq: Diyala**: Hamrin, near Shahraban, 34°16'06.8"N, 44°48'48.5"E, 8 May 1958, *s. col*. s,n. (E); Shahraban, 33°56'09.4"N, 44°55'10.8"E, 11 April 1957, *Haines Wheeler* 847 (E,K); Mandali, 33°42'48.9"N, 45°32'06.1"E, 26 March 1932, *E.R. Guest* 1742 (K, BAG); 30 km north east Mandali, 210 m, 33°48'35.5"N, 45°34'48.7"E, 26 April 1979, *Al-kaisi & Khayat* 50782 (K); **Basrah**: 77 km northwest of Zubair, 30°36'12.5"N, 47°00'36.7"E, 19 March 1964, *Fred Barkley & Hikmat Abbas Al-ani* 6499 (K,W); Near Jalibah, 30°27'57.0"N, 46°52'02.6"E, 8 April 1933, *s. col.* 5065 (K); 28 km south east by south of Zubair, 12 m, 30°16'33.4"N, 47°47'35.6"E, 23 March 1957, *E. R. Guest, A. Rawi & K. H. Rechinger* 16875 (K, BAG); 70 km east of Zubair, 30°17'35.3"N, 48°06'29.9"E, 13 February 1973, *Turner* 47457 (K); Between Zubair and Safwan, 30°15'41.7"N, 47°41'29.4"E, 23 March 1966, *H. Alizzi* 34341 (K); Rumaila, Toba railway station 20 km west of Ghubaishiyia, 30°32'56.0"N, 47°17'51.3"E, 27 March 1965, *Sharif Y. Haddad* 9535 (K); 30 km west of Jabal Sanam, 30°08'13.1"N, 47°27'36.6"E, 15 April 1963, *Khalid Alizzi* 32684 (K, BAG); Umm Qasr Port, 30°01'43.7"N, 47°56'05.3"E, 13 March 1973, *Husain Al-ali* 39929 (K); Jabal Sanam, 150 m, 30°07'43.5"N, 47°37'09.5"E, 6 March 1961, *s. col.* 29889 (BAG); Southern desert of Zubair, 30°20'00.0"N, 47°40'00.0"E, 23 March 1957, *K. H. Rechinger* 5247 (W); Shaib Al-batin, Jarishan, 30°04'06.1"N, 47°09'25.4"E, 24 March 1957, *K. H. Rechinger* (W); 6 km Southeast of Safwan, 30°05'08.2"N, 47°47'51.7"E, 23 March 1957, *K. H. Rechinger* 5245 (W); **Anbar**: 10 km N of Rutba, 33°09'48.3"N, 40°15'29.1"E, 28 February 1947, *Rawi & Gillett* 6326 (K); 10 km from Hit to Kubaysah, 33°38'19.9"N, 42°48'17.0"E, 85 m, 31 March 1976, *S. Omar, Alkaisi, K. Hamad & H. Hamid* 44354 (K); Ramadi east of Lake Tharthar, 33°29'28.6"N, 43°16'56.9"E, 3 April 1964, *Fred A. Barkley & Ramdan Eljumaili* 7263 (K); Shbaichan road 10 km north of Rawah, 34°34'24.8"N, 41°56'17.7"E, 260 m, 3 April 1962, *Khatib & Hlizzi* 31967 (K); 20 km north west Fallujah, 33°23'45.6"N, 43°48'58.9"E, 24 April 1982, *Omar & Alkhayat* 31967 (BAG); 10 km from Hit to Kubaysah, 33°38'19.9"N, 42°48'17.0"E, 85 m, 31 March 1976, *S. Omar, Alkaisi, K. hamad & H. Hamid* 44354 (BAG); Between Fallujah and Wadi Tharthar, 33°32'24.2"N, 43°37'13.0"E, 3 May 1957, *K. H. Rechinger* 11247 (W); **Muthanna**: 10 km south of Samawah, 31°15'03.0"N, 45°17'21.1"E, 20 m, 21 February 1947, *Rawi & Gillett* 6125 (K); 15 km west of Samawah, 31°18'44.5"N, 45°07'24.0"E, 20 m, 19 March 1955, *Ali Rawi* 14880 (K); 25 km to Busaiya from Al-Khidr Al-mai, 30°12'23.7"N, 46°20'46.6"E, 200 m, 24 February 1978, *Alkaisi, K. Hamad & H. Hamid* 48514 (K); Al-Khidr Al-mai enclosure, 31°12'00.7"N, 45°33'11.5"E, 21 January 1978, *F. Karim, A. Sharief, K. Hamad & H. Hamid* 48066 (K); 50 km east of Busaiya to Al-khidr Al-mai, 30°39'58.4"N, 46°01'13.1"E, 21 January 1978, *F. Karim, A. Sharief, K. Hamad & H. Hamid* 48034 (K); 13 km west Samawah, 31°19'42.8"N, 45°12'30.5"E, 40 m, 26 March, *Ibrahim Al-mahallal* 15204 (K,BAG); **Wasit**: Kut, 32°39'07.9"N, 45°45'49.2"E, 19 April 1967, *Alizzi & S. Omar* 34893 (K); 55 km east of Kut, 32°26'50.8"N, 46°23'40.4"E, 6 March 1963, *F. A. Barkley* 33Ir4055 (K); 5 km from Badra to Kut, 32°33'17.2"N, 45°48'13.6"E, 90 m, 12 March 1977, *Al-kaisi & H. Hamid* 46525 (K,BAG); 51 km northeast of Kut between Jassan and Badrah, 33°01'52.0"N, 45°54'14.1"E, 5 April 1964, *Hikmat Abbas & F. R. Bharucha* 2613 (K,W); 80 km west of Shayk Sa’d, 32°35'05.8"N, 46°08'01.5"E, 30 m, 4 April 1958, *Ali Rawi & S. Haddad* 25520 (K); 10 km east of Zurbatiyah, 33°11'38.8"N, 46°04'38.3"E, 240 m, 13 March 1977, *Al-kaisi & H. Hamid* 46551 (BAG); **Karbala**: 8 km west of Karbala, 32°31'20.8"N, 44°00'59.8"E, 9 March 1947, *Rawi & Gillett* 6415 (K); Razazza, 32°37'59.4"N, 43°53'52.1"E, 38 m, 18 March 2019, *A. Haloob, Ikhlas, R. Hamshkan & Riyadh* 59879 (BAG); 2 km west of Ukhaidir, 32°26'25.5"N, 43°35'30.1"E, 60 m, 12 March 1980, *s.col*. 51219 (BAG); 18 km west of Karbala, 32°33'18.5"N, 43°53'14.1"E, 40 m, 4 May 1964, *Martin L. Grant* 18228 (W); **Dhi Qar**: Eridu, 30°49'49.9"N, 45°59'54.3"E, 1 February 1947, *Seton Lloyd* 6328 (K,BAG); **Nineveh**: Faidah Al-rbaswi, 36°37'09.7"N, 42°58'38.3"E, 7 April 1973, *F. Karim, M. Noori, H. Hamid & H. Kadhim* 40279 (K); 6 km from Rabia, 36°47'17.3"N, 42°06'55.9"E, 1 April 1973, *F. Karim, H. Hamid & H. Kadhim* 39944 (K); **Najaf**: Al-Hira, 31°53'18.1"N, 44°29'28.6"E, 8 m, 6 March 2018, *Riyadh, Yasin, Dhya’a, Adel & Sinan* 59291 (BAG). FPF, chelat-Amara, AL-Mayah and AL-Asady 16122 BSRA. FPF, wadi AL-Tib -Mayah and AL-Asady 15130 BSRA. DSD, Basrah-Nassiria road, 30km from Zubair towords Nassirya, AL-Mayah 1995 BSRA. DSD, Slop of jabal sanam, s.w. of Safwan, Basrah, I.A.AL-Mayah and J.Dehry 1597,BSRA. DSD, Zubair, AL-Mayah and AL-Asady 1404 BSRA. DSD,JARISHAN AL-Mayah and AL-Asady 1418 BSRA. DSD, Jabal Sanam AL-Mayah and AL-Asady 1419 BSRA.

## Supplementary Material

XML Treatment for Cistanche tubulosa

## ﻿Additional information

### Author ORCIDs

Chris J. Thorogood https://orcid.org/0000-0002-2822-0182

Julie A. Hawkins https://orcid.org/0000-0002-9048-8016

### Data availability

All of the data that support the findings of this study are available in the main text.
